# Host suitability of Brassicaceae crops for *Belonolaimus longicaudatus* in greenhouse conditions

**DOI:** 10.2478/jofnem-2025-0029

**Published:** 2025-07-05

**Authors:** Sabina Budhathoki, Zane J. Grabau

**Affiliations:** Entomology and Nematology Department, University of Florida, 1881 Natural Area Drive, Gainesville, FL 32611, United States

**Keywords:** arugula, *Belonoloamius longicaudatus*, *Brassica carinata*, *Brassica juncea*, caliente mustard, carinata, *Crotalaria juncea*, *Eruca sativa*, host status, potato, sorghum-sudangrass, *Sorghum x drummondii*, sting nematode, sunn hemp

## Abstract

Sting nematode (*Belonolaimus longicaudatus*) acutely damages many vegetables in the Southeast U.S. Brassicas are known to suppress some plant-parasitic nematodes (PPNs), but the relationship of many brassicas with sting nematode has not been studied. This information would help growers in making decisions about using brassicas in rotation with vegetables in the region. Therefore, the objective of this study was to assess the host suitability of arugula (*Eruca sativa* ‘Nemat’), caliente mustard (*Brassica juncea* ‘Rojo’) and carinata (*Brassica carinata* ‘NJUET 400’) brassicas for sting nematode as compared to crops with known host status for sting nematode: a poor host, sunn hemp (*Crotalaria juncea* ‘Crescent Sunn’) and a good host, sorghum-sudangrass (*Sorghum x drummondii ‘*Defiance’). Repeated greenhouse experiments were conducted in 2024 with each treatment replicated 6 times. All brassicas — arugula, caliente mustard, and carinata — had higher final sting nematode abundances than sunn hemp and greater or similar abundances to sorghum-sudangrass. This, along with the reproductive factor > 1, indicated that the brassicas tested are good hosts for sting nematode and may not be suitable options for rotation where this nematode is present. However, field research is needed to verify this result and evaluate the impacts of these brassicas on subsequent cash crops.

Vegetable production in Florida is one of the state’s biggest industries, covering 93,726 ha of harvested land, with a total production value of $2.26 billion (USDA-NASS, 2023). Sting nematode (*Belonolaimus longicaudatus* Rau, 1958) is one of the major parasitic nematodes (PPN) of vegetables in Florida and the southeastern United States, and is found predominantly in sandy soils (Smart and Nguyen, 1991). It can damage a wide variety of vegetables, such as potato (*Solanum tuberosum*), cabbage (*Brassica campestris*), and strawberries (*Fragaria* x *ananassa*), even at low abundances (Kutsuwa et al., 2015; Watson et al., 2020). Sting nematode feeds on the root meristems, causing general stunting of the root system, along with the formation of stubby, proliferative lateral roots (Robbins and Hirschmann, 1974). In strawberry, the presence of a single sting nematode per field warrants management to prevent damage (Watson et al., 2020). In potato, two or three sting nematodes per 100 cm^3^ of soil can reduce the yield by 199 kg/ha (Crow et al., 2000). Given sting nematodes’ significant impact, effective control measures must be employed to mitigate yield losses and ensure sustainability of vegetable production.

Sting nematode management is challenging for growers. Currently, many growers rely heavily on the use of chemical nematicides, particularly the fumigant 1,3-dichloropropene (1,3-D), to manage sting nematodes (Grabau et al., 2019). However, nematicide options are limited and expensive, and there are increasing restrictions on the use of soil fumigants due to their harmful environmental impact (Abawi and Widmer, 2000). Furthermore, sting nematode-resistant cultivars are not available, making management efforts even more difficult (Grabau and Noling, 2019). Therefore, alternative management strategies are needed to effectively manage sting nematodes.

One such approach is incorporating cover crops into crop rotations for vegetable cropping systems to manage pests, diseases, and nematodes while also improving soil health (Norris and Congreves, 2018). Cover crops can manage PPNs by being non-hosts (with no reproduction) or poor hosts (with limited reproduction) for specific PPNs, producing toxic nematicide compounds, or serving as trap crops (Abd-Elgawad, 2021). This study focuses on investigating the host status of cover crops for sting nematode.

Cover crop host status varies by cover crop species and plant-parasitic nematode species. For instance, in northeast Florida, potatoes are often rotated with summer cover crops like sorghum-sudangrass, which is a good host of sting nematode (Weingartner et al., 1993). Another commonly grown summer cover crop in the Southeast is sunn hemp (*Crotolaria juncea*), which is known to be a poor host for many economically damaging PPNs (Marquez and Hajihassani, 2023). There are few studies on the effects of sunn hemp on sting nematodes, but there are some reports of this crop reducing sting nematode abundances in greenhouse or field conditions (Braz et al., 2016; Tsegay et al., 2024).

In vegetable systems in the southeastern U.S., summer cover crops are recommended to complement vegetables grown in cooler seasons (Wang et al., 2008; McSorley et al., 2009). Cool-season cover crops such as brassicas also have the potential to be incorporated along with summer cover crops in certain rotations, especially when vegetables are grown in the winter or spring. Brassicas are short-season winter crops that can act as poor or non-hosts for certain PPNs, releasing volatile, toxic compounds (isothiocyanates) from glucosinolates that act is a biofumigant to kill nematodes (Fourie et al., 2016; Dutta et al., 2019). Even when brassicas are used for biofumigation, selecting species that are poor or non-hosts to PPNs remains a key consideration. Previous studies have shown the variable quality of brassicas as hosts for certain PPNs such as *Meloidogyne* spp. For example, *Brassica juncea* ‘Nemfix’ and *B. rapa* ‘Samson’ are good hosts for most root-knot nematodes, including *Meloidogyne hapla*, *M. incognita* and *M. javanica*; *Eruca sativa* ‘Nemat,’ as well as *Raphanus sativus* ‘Boss’ and ‘TerraNova,’ are poor hosts to them (Edwards and Ploeg, 2014).

However, there are no studies examining the relationship between brassica cover crops and sting nematodes — an important gap in our knowledge. Because sting nematodes have a low economic threshold, growers often prioritize chemical nematicides over exploring cultural options, which may be one reason for this gap. Furthermore, in the United States, sting nematodes are problematic primarily in the Southeast (Watson and Desaeger, 2019; Grabau et al., 2023) where brassica cover crops have not been prevalent. However, there has recently been increased focus on brassicas in the Southeast as brassicas such as caliente mustard ‘Rojo’ and arugula ‘Nemat’ have been marketed for their potential nematode suppression capabilities. Specifically, caliente mustard ‘Rojo’ is marketed as containing high concentrations of glucosinolates, and arugula ‘Nemat’ can serve as a trap crop for certain root-knot nematode species (Melakeberhan et al., 2006).

Carinata is an emerging brassica winter oilseed crop in the southeast U.S. that is gaining increasing research and commercial interest for its use as a jet fuel (Seepaul et al., 2021). Carinata may help with reniform nematode management, as greenhouse studies demonstrated that it was a poor host, with plant residues that suppressed reniform nematode (Sandoval-Ruiz and Grabau, 2023a; 2003b), although results against reniform nematode were mixed in a rotation study under greenhouse conditions (Sandoval-Ruiz and Grabau, 2023c). However, Carinata’s effects on other PPNs, including sting nematode, remain unknown. Understanding the relationships between these brassicas and sting nematode is crucial in order to determine if these emerging crops could help manage sting nematodes. Because sting nematode is a major concern in the region, this may also influence grower adoption of these brassicas. Based on this need, the objective of this study is to evaluate the host suitability of different brassica cover crops for sting nematodes under greenhouse conditions.

## Materials and Methods

### Experimental design

Two repeated pot trials (Trial I and Trial II) were conducted at the Entomology and Nematology Department, University of Florida in Gainesville, Florida, in two closely-located greenhouses. Trial I was carried out from April to July 2024 and Trial II from May to August 2024.

The experiment was arranged in a Randomized Complete Block Design (RCBD) with six replications per treatment in each trial. The treatments consisted of different rotational crops: (i) sunn hemp ‘Crescent Sunn’ (Tropical Seeds LLC, Miami, FL); (ii) sorghum-sudangrass ‘Defiance’ (Kelly Seed Company, Hartford, AL); (iii) caliente mustard ‘Rojo’ (High Performance Seeds, Inc., Moses Lake, WA); (iv) arugula ‘Nemat’ (High Performance Seeds, Inc.); and (v) carinata ‘NUJET 400’ (Nufarm Americas Inc., Alsip, IL). Sunn hemp was selected as a non-host for sting nematode (Braz et al., 2016) and sorghum-sudangrass as a good host for sting nematode (Weingartner et al., 1993).

### Sting nematode inoculum preparation

Sting nematode inoculum used in the trials were collected from naturally-infested soil at the Hastings Agriculture and Education Center (29.693722, −81.443889), Hastings, FL, that has a history of sorghum-sudangrass and potato production. To establish a pure culture, 100 cm^3^ of field soil was suspended in water. The soil suspension was poured through a 400-mesh sieve and the nematodes were collected in a Baermann bowl, following the method of McSorley and Frederick (1991). The solution was incubated at room temperature for 48 hr to allow nematodes to migrate into the water. After that, the solution was poured through a 500-mesh sieve and rinsed, and the nematode suspension was collected in a beaker. Individual sting nematodes were picked from the solution using a metal thread guided by magnification under a Zeiss SteREO Discovery V8 microscope (Zeiss, Gottingen, Germany). Sorghum-sudangrass ‘Defiance’ was planted in about 20 to 25 pots, and 100 sting nematodes were inoculated into each pot. The plants were maintained in the greenhouse for four to five months to increase population density.

To inoculate trials, soil from the sorghum-sudangrass pot containing pure sting nematode culture was extracted, following the procedure for establishing sting nematode cultures from field soil described above. The sting nematode inoculum was then quantified using a Primovert inverted microscope (Zeiss, New York, USA). Sting nematodes were inoculated into each experimental pot on the same day the inoculum was extracted.

### Trial establishment and maintenance

Seeds of the cover crops were sown on 22 April 2024 for Trial I and 22 May 2024 for Trial II. The soil used for planting was collected from the University of Florida’s North Florida Research and Education Center, located near Live Oak, FL (30.304769, −82.897703). This soil is classified as a Chipley-Foxworth-Albany complex (91% sand, 6.8% silt, 2.4% clay, and 1.7% organic matter) (USDA-NRCS, 2019). The soil was autoclaved at 121 °C for 90 minutes using an Amsco Lab 250-LV autoclave (Mentor, OH) and stored before it was used for planting. Clay pots with a 15-cm diameter were filled with 1,000 cm^3^ of the autoclaved soil. Six seeds were initially planted in each pot, but two weeks later, the seedlings were thinned to one per pot. Sting nematodes were inoculated onto plants at the two-to-three-true-leaf stage of plants, which was 18 days after planting (DAP) in Trial I and 25 DAP in Trial II. For sting nematode inoculation, four holes 2.5 cm deep were made around each plant, and the nematode inoculum was evenly distributed into the holes using a pipette. Each pot received a total of 100 mixed stages of sting nematode juveniles and adults in 4 mL of solution. Plants were maintained and terminated at 60 days for Trial I and 58 days for Trial II to ensure sufficient time for crop establishment and sting nematode infection. Trial establishment, data collection and crop management details are given in Table 1.

**Table 1:** Schedule of plant data collection, nematode inoculation and crop growth ratings in greenhouse trials assessing crop host status for sting nematode.

**Activities**	**Dates**			
	**Trial I**	**DAI^a^**	**Trial II**	**DAI**
Seeding	22 April 2024	−18	22 May 2024	−25
Thinning	6 May 2024	−4	6 June 2024	−11
Sting nematode inoculation	10 May 2024	0	17 June 2024	0
First crop growth assessment	10 June 2024	30	17 July 2024	30

a DAI indicates days after inoculation. Negative values indicate days before inoculation.

Plants were fertilized with Miracle-Gro (Scotts Miracle-Gro Products, Inc., Marysville, OH) once after thinning and hand-watered each day. No supplemental lighting was provided during the trials. Greenhouse temperatures were recorded using a HOBO MX TidbiT 400 (Onset Computer Corporation, Bourne, MA), with average temperatures of 29.87 °C (maximum: 46.99 °C, minimum: 18.54 °C) in Trial I and 28.33°C (maximum: 47.33 °C, minimum: 19.55 °C) in Trial II.

### Nematode quantification and plant growth assessment

Plant growth parameters, including plant height and number of leaves per plant, were recorded 30 days after nematode inoculation (DAI) and just prior to termination. At termination, the shoots from each pot were clipped above the soil line, and the fresh-shoot weights of each cover crop were measured separately. The roots were gently removed from the pot and shaken into a beaker to remove excess soil particles. The roots were then carefully washed to remove the remaining soil, and a blotting paper was used to absorb the surface water. Finally, the roots were weighed.

For soil nematode extraction, the soil from each pot was thoroughly mixed, and 100 cm^3^ soil was extracted using the sucrose centrifugation method (Jenkins, 1964). Sting nematodes were quantified by morphological identification under an inverted microscope. Additionally, the reproduction factor of sting nematode (RF = Final abundance/Initial abundance) was calculated for each pot. The final population of sting nematodes represents the total number quantified in 1,000 cm^3^ soil, extrapolated from an initial 100 cm^3^ sample. The initial abundance indicates the total number of sting nematodes inoculated per pot, which was 100. In nematology research, the reproduction factor is used as an indicator of suitability of a host plant for the nematode; a plant with RF > 1 is considered a good host plant, RF < 1 indicates a poor host, and RF = 0 a non-host plant (Seinhorst, 1967). The ratio of sting nematodes to root biomass was also calculated by dividing the total number of sting nematodes extracted from soil by fresh root weight in grams for each pot.

### Statistical analysis

The analysis was done using R version 4.1.1 (2021). Most variables had trial-by-treatment interactions (ANOVA, *P* ≤ 0.05), and therefore the data were analyzed separately by trial. The normality and homogeneity of variance of all variables was assessed using diagnostic plots of residuals versus predicted value (Schützenmeister et al., 2012) and further validated using the Shapiro-Wilk test for normality and Levene’s test for homogeneity of variance. Variables that did not meet these tests were subjected to square root transformation. All variables at all time points were subjected to square root transformation, except for plant height and leaf number, which were never transformed. One-way ANOVA was performed for all the variables to determine the differences among treatments. For each variable, if the treatment effects in ANOVA were significant (*P* ≤ 0.05), Tukey’s HSD test was conducted to separate the means (α = 0.05). Statistical analysis was conducted on the transformed means as applicable, but only true means are presented in tables and figures.

## Results

### Host status of crops to sting nematode

**Figure 1:**
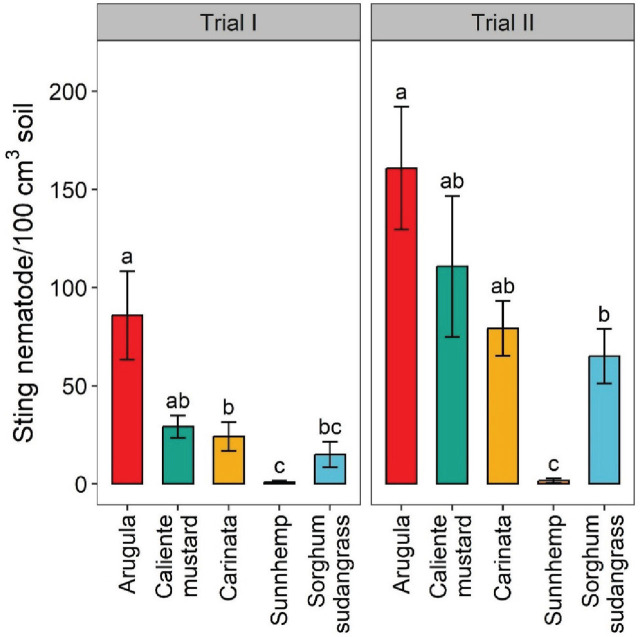
Effects of different crop treatments on final soil sting nematode abundances in greenhouse trials. Values are means (N = 6) with error bars indicating standard errors. Within a trial, means that share a letter are not significantly different based on Tukey’s HSD (*P* < 0.05).

Sting nematode soil abundance was significantly greater for arugula than for sunn hemp, by 99%, in both trials. It was 83% greater for arugula than for sorghum-sudangrass in Trial I and 60% greater in Trial II (Fig. 1). In both trials, sting nematode soil abundances were significantly greater for caliente mustard and carinata than for sunn hemp, by 97%–98%. However, caliente mustard and carinata had sting nematode soil abundances similar to sorghum sudangrass in both trials. Among brassicas, there were no significant differences in sting nematode soil abundances, except that abundances were greater for arugula than for carinata in Trial 1. Sunn hemp supported the lowest sting nematode soil abundances of any crop in both trials, similar only to sorghum-sudangrass in Trial I. Sting nematode abundances generally were numerically greater in Trial II compared to Trial I across all crops tested.

**Figure 2:**
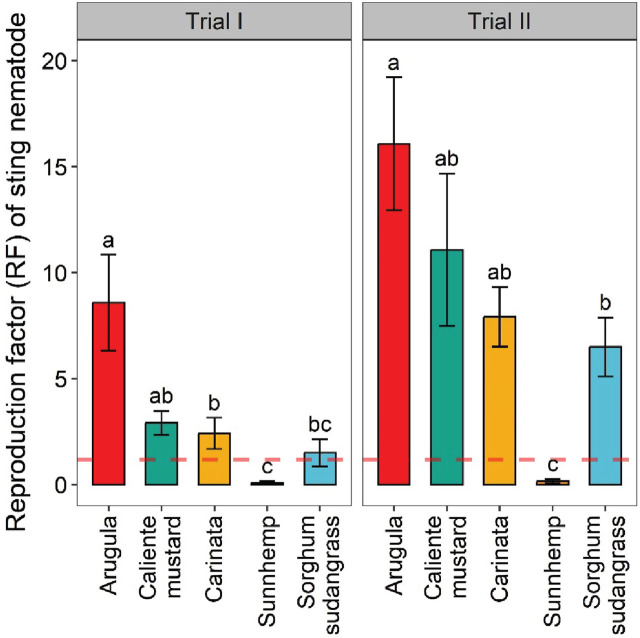
Effects of different crop treatments on sting nematode reproduction factor in greenhouse trials. Reproduction factor (RF) is final sting nematodes population per pot/inoculated initial population; Dashed line indicates RF = 1. Values are means (N = 6) with error bars indicating standard errors. Within a trial, means that share a letter are not significantly different based on Tukey’s HSD (*P* < 0.05).

Reproduction factor (RF) varied significantly among crops (Fig. 2), and trends were similar to those described for sting nematode soil abundances. Each brassica and sorghum-sudangrass crop had RF > 1, indicating that it was a good host for sting nematode. Arugula had the greatest RF, with values of 8.58 in Trial I and 16.08 in Trial II. The RF of caliente mustard and carinata were significantly greater than that of sunn hemp, but similar to sorghum-sudangrass, in both trials. The RF of caliente mustard was 2.91 in Trial I and 11.08 in Trial II, while carinata’s RF was 2.41 in Trial I and 7.91 in Trial II. Sunn hemp had RF < 1, with values of 0.08 in Trial I and 0.16 in Trial II, indicating that it is a poor host for sting nematode.

### Ratio of sting nematode soil abundances to root biomass

**Figure 3:**
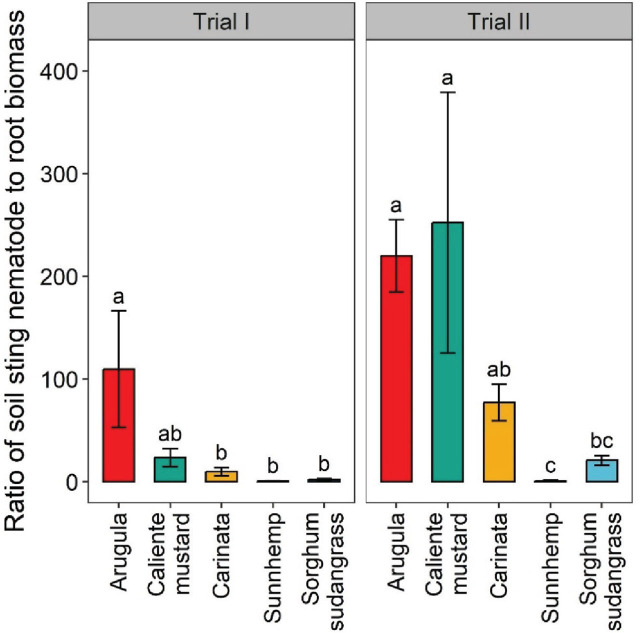
Ratio sting nematode soil abundances to root biomass in greenhouse Trial I and Trial II. Values are means (N = 6) with error bars indicating standard errors. Within a trial, means that share a letter are not significantly different based on Tukey’s HSD (*P* < 0.05).

In Trial I, arugula significantly increased the ratio of sting nematode soil abundances to root biomass by 91%, 99%, and 98% relative to carinata, sunn hemp, and sorghum-sudangrass, respectively (Fig. 3). The sting nematode to root biomass ratio was intermediate for caliente mustard, not differing significantly from any of the other crops in Trial I. In Trial II, however, the sting nematode to root biomass ratio significantly increased in both arugula and caliente mustard — by 100% compared to sunn hemp, and by 91%–92% compared to sorghum-sudangrass. For carinata in Trial II, however, sting nematode to root biomass ratios were intermediate, not significantly differing from arugula, caliente mustard or sorghum-sudangrass. Sunn hemp supported the lowest ratio of sting nematodes to root biomass of all crops, though it was not different from sorghum-sudangrass in either trial, or from carinata in Trial I.

### Variation in vegetative growth among crops

**Figure 4:**
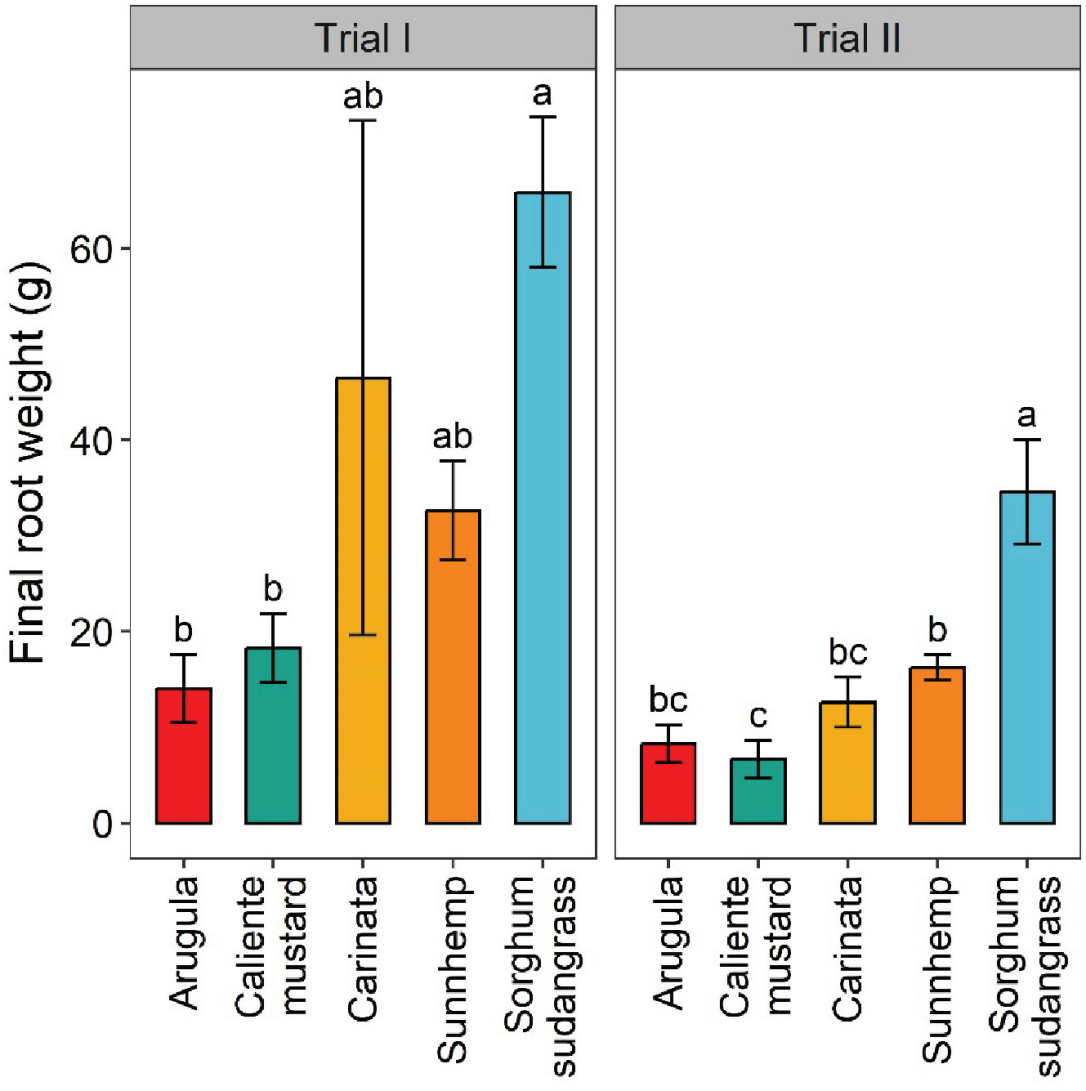
Final fresh-root weight of crops at termination of greenhouse trials. Values are means (N = 6) with error bars indicating standard errors. Within a trial, means that share a letter are not significantly different based on Tukey’s HSD (*P* < 0.05).

Sorghum-sudangrass had significantly greater root biomass in trial I, with an increase of 79% over that of arugula and 72% over that of caliente mustard (Fig. 4). Sorghum-sudangrass also had significantly greater root biomass than any other crop in Trial II.

**Figure 5:**
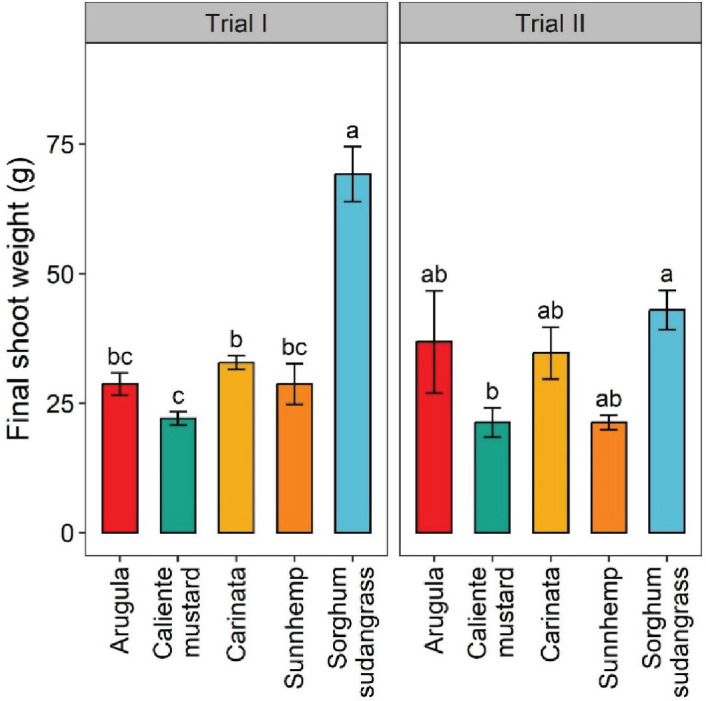
Final shoot weight of crops at termination for greenhouse Trial I and Trial II. Values are means (N = 6) with error bars indicating standard errors. Within a trial, means that share a letter are not significantly different based on Tukey’s HSD (*P* < 0.05).

Root biomass was significantly greater, by 59%, for sunn hemp than for caliente mustard in Trial II. Fresh shoot weight was significantly greater for sorghum-sudangrass than any other crop in Trial I, but only greater than caliente mustard in Trial II (Fig. 5). Fresh shoot biomass was also significantly greater for carinata than caliente mustard, by 33%, in Trial I. For both trials and sampling dates, plant heights were generally greatest for sorghum-sudangrass, followed by sunn hemp, caliente mustard, carinata, and then arugula (Table 2). Sunn hemp also had greater leaves per plant than any other crop in both trials and sampling dates, whereas sorghum-sudangrass had the fewest leaves at crop termination (Table 2).

**Table 2:** Plant height (cm) of different crop treatments in greenhouse trials.

	**Plant height**			
	**Trial I**		**Trial II**	
Crops	30 DAI^a a,b^	60 DAI	30 DAI	58 DAI
Arugula	15 d^b^	18 d	26 d	23 d
Caliente mustard	80 b	116 ab	53 bc	82 bc
Carinata	39 c	69 c	34 cd	53 c
Sunn hemp	65 b	93 bc	60 b	92 b
Sorghum-sudangrass	148 a	131 a	139 a	152 a
*P*-value	^***^	^***^	^***^	^***^

	**Number of leaves**			
	**Trial I**		**Trial II**	
Crops	30 DAI ^a,b^	60 DAI	30 DAI	58 DAI
Arugula	14 b	24 b	24 b	24 b
Caliente mustard	16 b	27 b	16 b	25 b
Carinata	14 b	27 b	11 b	15 bc
Sunn hemp	40 a	52 a	43 a	54 a
Sorghum-sudangrass	9 b	11 c	10 b	11 c
*P*-value	^***^	^***^	^***^	^***^

a DAI indicates days after inoculation.

b Values are means. Letters within each DAI indicate significant differences according to Tukey’s HSD (*P* < 0.05).

‘***’ indicates ANOVA with *P* ≤ 0.01.

## Discussion

All the brassicas tested were good hosts of sting nematodes, with RF > 1, which is the established threshold for host status (Seinhorst, 1967). Comparisons with cover crops that are known hosts also support this conclusion, as arugula supported greater sting nematode soil abundances than either sunn hemp — a poor host — or sorghum-sudangrass — a good host crop (Weingartner and Shumaker, 1990). Caliente mustard and carinata also supported greater sting nematode populations than sunn hemp but at a level similar to sorghum-sudangrass. To our knowledge, this is the first study to evaluate the host suitability of brassicaceous rotational crops for sting nematode. Therefore, the findings from this study will be relevant to protecting crops and improve cropping systems in the southeastern U.S., where sting nematode is prevalent. The results of this study complement previous studies where brassicaceous crops, such as kale (*Brassica oleracea* var sabellica), collard (*Brassica oleracea* var acephala), cauliflower (*Brassica oleracea* var botrytis), and cabbage, are reported to be susceptible to sting nematode damage, though host status was not reported based on RF values in those studies (Rhoades, 1971; Khuong and Smart, 1975).

Sunn hemp and sorghum-sudangrass impacts on sting nematode were consistent with a poor host and good host, respectively, as reported in previous studies. Sorghum-sudangrass supported greater sting nematode abundances in this study and was a good host for sting nematode, similar to findings in previous studies (Rhoades, 1983; Weingartner et al., 1993; Crow et al., 2001). Therefore, sorghum-sudangrass is detrimental to sting nematode management. In this study, sunn hemp substantially lowered sting nematode abundances, indicating that it was a poor sting nematode host, as previously found (Braz et al., 2016; Grabau et al., 2022; Tsegay et al., 2024). Overall, this supports prior findings that sunn hemp is a good choice as a rotation crop in sting nematode-infested areas, and sorghum-sudangrass is a poor choice.

Overall, the data from this research indicate that the brassicas arugula, caliente mustard, and carinata are not suitable cool-season crops to use in rotation where sting nematode is a problem. Based on results in the present greenhouse study, these brassicas would be expected to increase sting nematode abundances in field production, leading to yield reductions in susceptible cash crops in the southeastern U.S. This increased sting nematode pressure may lead to increased crop management costs for growers as well. Field research is necessary to verify this.

On the other hand, this study also shows the potential of sunn hemp as a summer cover crop for sting nematode management. Sunn hemp may help minimize sting-nematode pressure for the susceptible vegetables and improve their yield. This information also provides a foundation for establishing sunn hemp as a summer rotational crop in sting nematode-prevalent regions such as northeastern Florida, where susceptible crops like potato and cabbage are grown. Sunn hemp also fixes atmospheric N, which can supply soil nutrients and can reduce fertilizer use (Shekinah and Stute, 2018). Therefore, sunn hemp should serve as a good summer cover crop in vegetable cropping systems in the southeastern U.S.

One limitation of this study is that only a single sting nematode population and a single cultivar of each crop was tested. In the southeastern U.S., the pathogenicity profile — the range of susceptible crops — is known to vary among *B. longicaudatus* populations from different locations. For example, in a greenhouse study, *B. longicaudatus* isolates from Virginia were pathogenic to peanut, while those from Georgia and Florida were not (Perry and Norden, 1963). However, both Florida and Georgia populations affected peanut in other greenhouse studies (Robbins and Barker, 1973; Han and Dickson, 2004).

Similarly, in another greenhouse study, the pathogenicity profile varied among *B. longicaudatus* populations from different regions within Florida. The “Gainesville population” reproduced on strawberry and tomato but not on rough lemon, while the “Sanford population” affected tomato but not strawberry or lemon (Abu-Gharbieh and Perry, 1970). Furthermore, there are genetic and morphological differences among populations of *B. longicaudatus* such that this group may actually constitute multiple species (Habteweld et al., 2021). This may account for the variations in pathogenicity profiles among populations of this nematode. Therefore, the results of this study are most relevant for northern Florida, where the test sting nematode population was collected. Further testing using sting nematode populations from a range of locations would improve confidence about the applicability of results across the region.

Further, even for a single sting nematode population, responses can vary among different cultivars or accessions of a given crop. For example, although all accessions in a previous greenhouse study were poor hosts of sting nematode, different sunn hemp accessions showed variability in their final sting nematode abundances, ranging from 0 to 13 per 100 cm^3^ of soil (Braz et al., 2016). Variation in cultivar responses of sting nematode were also reported in pearl millet, where ‘HGM-100’ was a better host than ‘TifGrain 102’ (Timper and Hanna, 2005). Therefore, further testing would be needed to determine if responses to sting nematode are the same across different cultivars of the brassica species tested. In this study, only a single cultivar for each brassica species — carinata ‘NUJET 400’, arugula ‘Nemat’, and Caliente mustard ‘Rojo’ — were tested because of their relevance for growers in the southeastern U.S.

The variation in RF between Trial I and Trial II — despite similar average, minimum and maximum temperatures — may be due to other unmeasured variables, including soil moisture. In both trials, plants were hand-watered, which may have caused inconsistencies in soil moisture levels across the trials. Robbins and Barker (1974) reported that reproduction of *B. longicaudatus* is greatly influenced by soil moisture.

Field research is necessary to verify the influence of brassicas on sting nematode management. In greenhouse-controlled pot experiments, where plants grow without competition and minimal stress, RF values may not accurately reflect field conditions. For instance, in this study, sorghum-sudangrass had an RF of 5 in Trial II, which is almost twice the RF reported by Crow et al. (2001) in northern Florida field conditions (RF = 1.8). While environment and crop variety may contribute to this difference, evaluating these factors under field conditions is essential. Furthermore, in this study, crops tested were not incorporated into soil, which is the typical practice in commercial production. Incorporating residues may help suppress nematode abundances (Sandoval-Ruiz and Grabau, 2023b) — for example, through the release of biofumigant compounds. Residues were not incorporated in this study because the objective was to evaluate host status independent of any biofumigant effects. Incorporating brassica shoot residues to evaluate potential biofumigant effects on sting nematode is recommended for future research. While brassicas increased sting nematode abundances in this greenhouse study, field testing is also important for verifying how this affects subsequent cash crops, since improving cash productivity is the primary purpose of brassicas as rotation crops.

In conclusion, this study indicated that brassica species arugula ‘Nemat,’ caliente mustard ‘Rojo’ and carinata ‘NJUET 400’ are good sting nematode hosts and thus are poor options to use in rotation where sting nematode is present. Sunn hemp as a summer cover crop is a better option for managing sting nematode infestations. Future research on brassica cover crops for sting nematode management should focus on evaluations of different brassica species and cultivars, as well as sting nematode populations from multiple locations. While greenhouse studies provide insights into nematode host relationships, future field research is needed to assess the impact of rotational brassica crops on sting nematode abundances and yield of the subsequent cash crop.
